# Supplementary material for: Integrated Proteomics and Metabolomics Link Acne to the Action Mechanisms of Cryptotanshinone Intervention
Source: Front Pharmacol. 2021 Sep 1;12:700696. doi: 10.3389/fphar.2021.700696 (PMC8440807; doi:10.3389/fphar.2021.700696)
Supplement: Supplementary file 1 [file DataSheet1.ZIP › Supplementary table 1-10 and material 1-3/Material 3-Metlib-MSMS/POS-Metlib-MSMS/Metlib-MSMS/M831T137_1_forward/0.997,1,2-dioleoyl-sn-glycero-3-phosphatidylcholine,(M-H+2Na)+.pdf]

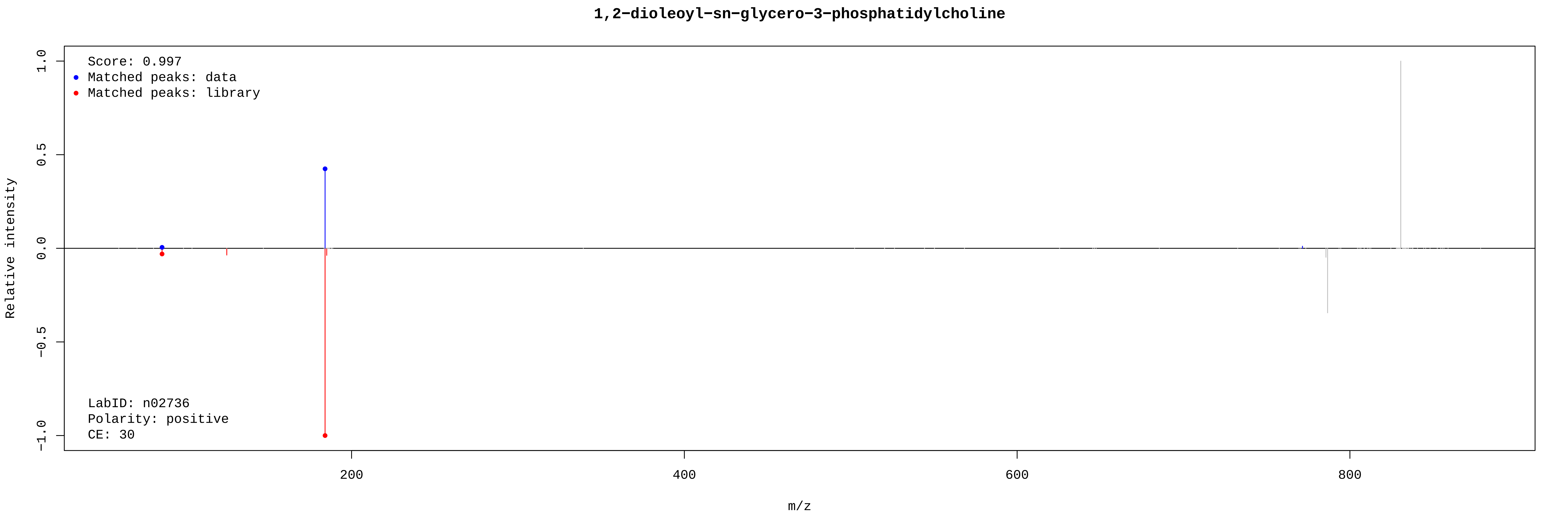
1,2-dioleoyl-sn-glycero-3-phosphatidylcholine
Score: 0.997
Matched peaks: data
Matched peaks: library
LabID: n02736
Polarity: positive
CE: 30
Relative intensity
1.0
0.5
0.0
-0.5
-1.0
200
400
600
800
m/z
